# High Cryptococcal Antigenuria Prevalence in a Population of PLHIV with Neurological Symptoms Hospitalized in the Infectious Diseases Wards of the Centre Hospitalier Universitaire de Libreville, Gabon

**DOI:** 10.3390/tropicalmed9120312

**Published:** 2024-12-23

**Authors:** Roger Hadry Sibi Matotou, Denise Patricia Mawili-Mboumba, Charlène Manomba, Bridy Chesly Moutombi Ditombi, Coella Joyce Mihindou, Dimitri Ardin Moussavou Mabicka, Arsène Mounomby, Solange Nzenze Afene, Marielle Karine Bouyou Akotet

**Affiliations:** 1 Department of Parasitology, Mycology and Tropical Medicine, Université des Sciences de la Santé (USS), Libreville BP 4009, Gabon; sibiroger617@gmail.com (R.H.S.M.); dpmawili@gmail.com (D.P.M.-M.); bridymoutombi@hotmail.com (B.C.M.D.); micojo93@yahoo.com (C.J.M.); dimitrimabicka7@gmail.com (D.A.M.M.); mounombyarsene@gmail.com (A.M.); andeme.solange@yahoo.fr (S.N.A.); 2 Department of Internal Medicine, Division of Infectious Diseases, Université des Sciences de la Santé (USS), Libreville BP 4009, Gabon; manomba20@gmail.com

**Keywords:** PLHIV, *Cryptococcus*, antigen, urine, Gabon

## Abstract

*Introduction:* Cryptococcal meningitis is a major cause of death in HIV/AIDS patients due to the existence of *Cryptococcus neoformans* in the central nervous system. Our objective was to evaluate the prevalence of Cryptococcus antigenuria in a population of HIV-infected patients in Libreville, Gabon. *Patients and Methods*: This study was conducted from April to October 2021 at the Infectious Diseases ward of the Centre Hospitalier Universitaire de Libreville. Hospitalized patients with HIV were included. The detection of cryptococcal antigen (CrAg) in urine was performed using the Pastorex Crypto Plus Kit. *Results*: Out of the 255 PLHIV, 142 benefited from the CrAg detection. The prevalence of urine CrAg was 24.6% (n = 35). The majority of CrAg+ patients (82.8%; n = 29) were under 55 years old. Almost three-quarters of them (n = 25; 71.4%) had CD4 counts < 200, and 80.0% (n = 28) were at WHO clinical stages III and IV. All patients with neck stiffness at admission had a CrAg positive test. *Conclusion*: This study showed a non-negligible prevalence of Cryptococcal urinary antigen in HIV-infected patients with neurological symptoms. These data underline the importance of CrAg screening in routine care for better management of PLHIV.

## 1. Introduction

Cryptococcal meningitis (CM) is a major cause of death in HIV/AIDS patients [1]. This disease caused by *Cryptococcus neoformans* (*C. neoformans*), damages the central nervous system, resulting in cryptococcal meningoencephalitis. Each year, nearly 112,000 deaths are caused by HIV-associated cryptococcal meningitis worldwide. Most of these occur in Sub-Saharan African countries [2,3]. Indeed, CM-related mortality in Africa is still at an unacceptably high level [4]. The mortality rate of cryptococcal meningitis has been estimated at 55% in low- and middle-income countries and at 20% in high-income ones [4,5,6].

It is an important etiology of death among people with advanced human immunodeficiency virus (HIV) disease [7]. The advent of antiretroviral therapy (ART) has significantly reduced the occurrence of cryptococcal meningitis in people living with HIV (PLHIV). However, CM contributes to high mortality even in patients under antiretroviral therapy in low-resource settings [8]. The clinical course of patients with asymptomatic antigenemia remains uncertain, even in patients receiving ART treatment. Nevertheless, if left untreated, the disease may develop as the fungal load increases in case of persistent immunodeficiency.

Prevention of the disease through routine screening (for subclinical infections), using cryptococcal antigen (CrAg) tests, and administration of preventive treatment need to be considered [1]. Indeed, mortality related to CM may be reduced based on the testing of HIV-infected people for *Cryptococcus neoformans* antigen detection [3] and the treatment strategy, as recommended by the World Health Organization (WHO) [9,10].

Thus, screening for cryptococcal antigen (CrAg) in patients enrolling in ART programs allows for the identification of patients at risk of cryptococcal meningitis; CrAg detection is also recommended for hospitalized PLHIV, mainly those in advanced disease. The diagnosis of CM is based on a microscopic direct examination of cerebrospinal fluid (CSF) culture to identify *C. neoformans*. The capsular antigen of this yeast can be detected using lateral flow assays but also with latex agglutination Cryptococcal antigen in blood and CSF prior to the onset of symptomatic meningitis. Furthermore, individuals with cryptococcal antigenemia are at higher risk of death compared to those without antigenemia, although they are under antifungal therapy [7,9].

Screening for cryptococcosis may also be achieved using urine samples. This may be a valuable and non-invasive method, which can be easily introduced in resource-limited settings. While lateral flow assays are widely used and have better sensitivity than latex agglutination assays, WHO does not strictly recommend one specific except CrAg [3]. Urine CrAg screening was shown to have a better sensitivity compared to serum antigen testing using latex agglutination [11].

In Gabon, the prevalence of cryptococcal meningitis is still not well known because of the scarcity of data. It was estimated at nearly 5% [12,13]. Nevertheless, in the aforementioned country, there is neither a formal recommendation or algorithm related to Cryptococcosis prevention nor management regarding CrAg detection. In some African settings, the screening for CrAg and “preemptive” treatment with fluconazole has been adopted [14]. Such a strategy could help better manage HIV-advanced diseases in remote areas, where there is little access to even microscopic equipment and where experienced trained technicians are lacking. Moreover, contra-indications of lumbar puncture are also frequent in CM-suspected patients. Therefore, serum or urine CrAg detection, which does not require additional material than the ones used for the hematological and biochemical testing for PLHIV routine follow-up, could be implemented in HIV ambulatory treatment centers and regional health structures where CSF culture is not routinely performed. The Pastorex Crypto Plus (Bio-Rad) is the first CrAg detection rapid test introduced in Gabon in 2021, and it is still the only CrAg test available in the country now. Generated data on its usefulness for CM diagnosis in hospitalized PLHIV with advanced HIV disease are necessary for a wide introduction and wide use of CrAg testing by the physicians and wide implementation by the policymakers.

The objective of this study was to estimate the prevalence of urinary cryptococcal antigen and the factors associated with the CrAg presence in a population of PLHIV hospitalized in the Infectious Diseases Ward of the Centre Hospitalier Universitaire de Libreville in Gabon, where data on the burden of Cryptococcal disease are still scarce.

## 2. Patients and Methods

### 2.1. Study Period and Site

A prospective cross-sectional study was conducted from April to October 2021 at the Centre Hospitalier Universitaire de Libreville (CHUL) in Gabon. The biggest healthcare structure in the country, CHUL, is a tertiary-level referral and teaching hospital that contains the main infectious diseases ward. In Libreville, where 857,000 inhabitants live, the prevalence of PLHIV is less than 10% [15].

### 2.2. Study Population

#### 2.2.1. Sample Size Determination

The sample size was calculated using the unique finite population. It was estimated by considering the prevalence of cryptococcal antigenemia in a Sub-Saharan African country (4.1%), and the technique employed is similar to the one performed in the present study [11]. Therefore, a minimum of 61 urine samples were estimated. Assuming that almost 20% will not provide a urine sample, the final minimum of CrAg tests to be performed would be 72.

#### 2.2.2. Participant Selection

Participants were included using a consecutive sampling approach. Thus, PLHIV participants were approached if they were aged above 18 years old and hospitalized in the CHUL infectious diseases ward with subjective symptoms of neurological cryptococcosis such as neck stiffness, mental confusion, convulsions, fever, headache, and altered consciousness. Those with a history of antifungal treatment or a confirmed diagnosis of cryptococcosis were not selected. After being informed about this study and after signing an informed consent form, each participant was interviewed by a team physician. Socio-demographic data (age, sex, marital status), date of HIV diagnosis, and antiretroviral treatment (ART) were recorded. Based on patient clinical and biological checking, comorbidities, size, weight, symptoms on admission, CD4+ cell count, and HIV WHO stage were also recorded on a standardized form for each patient [9,16]. The body mass index (BMI) was calculated; each patient had a unique code for identification.

After the interview, each participant was given a clean and sterile urine container, identified with the patient’s unique identification code, for urine specimen collection in the morning. Containers were transferred to the laboratory in an icebox for processing within two hours following urine collection.

Urine cryptococcal antigen detection (CrAg) was achieved using the Pastorex Crypto plus (Bio-Rad) in the Department of Parasitology, Mycology, and Tropical Medicine of the Université des Sciences de la Santé (USS, Gabon), as described elsewhere. Briefly, this qualitative and semi-quantitative agglutination, which detects the capsular glucuronoxylomannan polysaccharide of *C. neoformans* in biological samples, including urine, was used according to the manufacturer’s instructions. First, the urine was diluted in a glycine buffer. Afterward, 40 μL of the sample was added on a circle on the agglutination map and mixed with a drop of *Cryptococcus* latex, which is a latex particle sensitized with an anti-glucuronoxylomannan monoclonal antibody (mouse), in a glycine buffer. Negative control was gained by mixing 40μl of dilution buffer with a drop of latex. The card was then placed on a shaker for 5 min (160 rpm) at room temperature (+14–30 °C). The agglutination resulted from the reaction between the particles and glucuronoxylomannan and was visible to the naked eye. The results were obtained after five minutes. The quality control of the test was assessed using the internal control introduced by the manufacturer in the procedure. Thus, the sensitivity of the test was checked based on the detection of glucuronoxylomannan polysaccharide of Cryptococcus (200 ng/mL).

Statistical analysis: Data were collected in Microsoft Excel by double entry procedure. Statistical analyses were performed using Stat view 05. Differences between groups were assessed using chi2 or Fisher exact test. All reported *p*-values were two-tailed, and a *p*-value < 0.05 was considered statistically significant. For the calculation of the risk factors associated with antigenuria, crude odd ratios with confidence intervals were determined.

Ethical considerations: This study was approved by the scientific and ethics committee, which was the legal entity to authorize research activities during the COVID-19 epidemic phase, under the number PROT00064/2020/P/CSCOVID/P. Authorization was also obtained from the Ministry of Health and the CHUL Director of Medical Affairs. In this study, for anonymity, the participants received an identification code. A written informed consent was obtained from each patient. All tests were free of charge, and the results were also sent to the ward physicians for patient-appropriate management, according to the national recommendations.

## 3. Results

**Figure 1:**
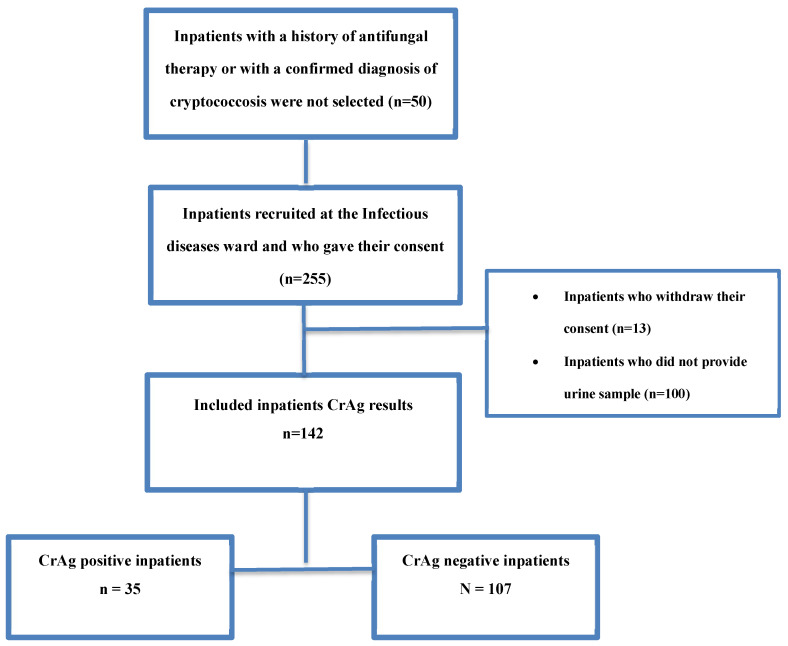
Flow chart of the study participants.

Out of 255 interviewed patients, 142 who agreed to participate in this study provided their urine samples (Figure 1). Almost three-quarters (n = 102; 71.8%) of them were female; the majority were unmarried (83.7%), worked (66.2%), and were on ART (Table 1). The median age was 42.5 [16.6–22.7] years. Among the patients with CD4 cell counts (n = 124), the median CD4 count level was 166 [100–300] cells/µL. Overall, 63.7% of them had a CD4 cell count below 200/µL (Table 2). In addition, the median BMI was 18.6 [16.6–22.7] kg/m^2^, and 66 (46.5%) were underweight. The median time of HIV diagnosis was 5 [1–11] years. According to the WHO clinical stages for HIV infection, patients were mainly at stages III and IV (n = 74; 52.1%), and 55 (38.7%) were at stage II. Overall, 28 (19.7%) participants had a comorbidity, which was mainly tuberculosis (Table 1). The frequent clinical signs at admission were fever (86.6%) and headaches (80.9%). Vomiting, altered consciousness, and seizures were observed in 7 (4.9%) patients; 7 had neck stiffness (4.9%), and 12 (8.4%) had respiratory symptoms.

### Frequency of CrAg Positive Test

All participants with CSF samples tested with detected *Cryptococcus neoformans* using India ink microscopic technique (n = 20) were also found Crag-positive. Only one participant who withdrew his consent was found CrAg-negative and microscopically positive. The Pastorex urine CrAg sensitivity was 95.2%, and its specificity was 87.6%. Thus, the prevalence of CrAg-positive tests was 24.6% (n = 35). No difference was found according to age, gender, matrimonial status, weight, school attendance, and ART use (Table 1.)

**Figure 2:**
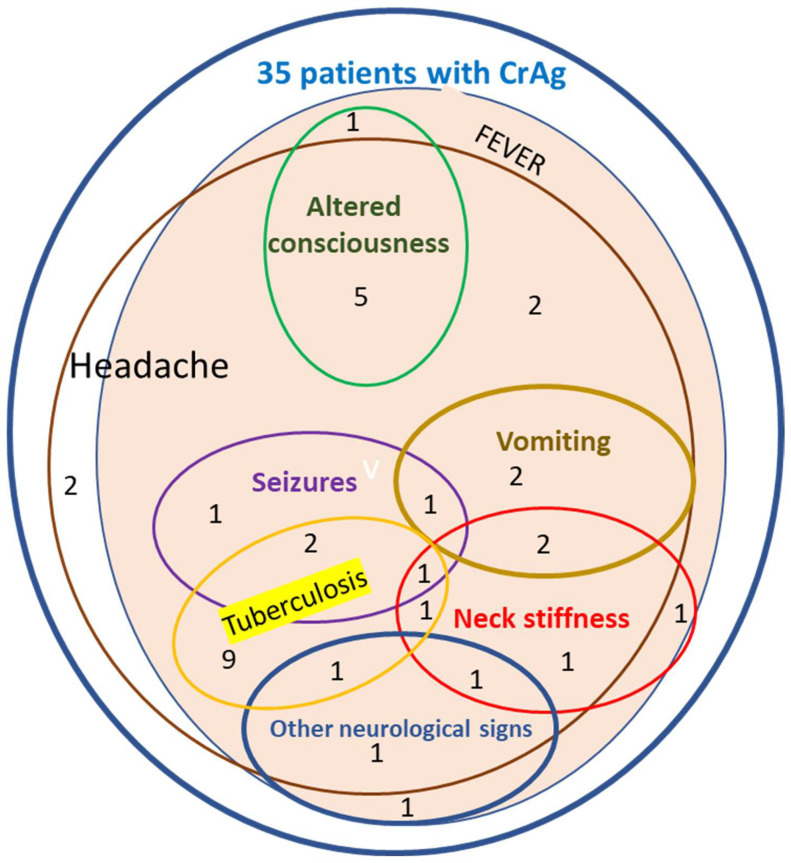
Clinical symptoms distribution in patients with positive urinary CRAg.

Clinical and immunological characteristics according to the presence of urine CrAg are reported in Figure 2 and Table 2. Tuberculosis (OR = 5.3 [95% CI: 2.1–13.1] *p* ˂ 0.01), a WHO stage III or IV (OR = 3.0 [95% CI: 1.2–7.5] *p* = 0.02), altered consciousness (OR = 21.7 [95% CI: 2.4–87.7] *p* ˂ 0.01), seizures (*p* ˂ 0.01), the presence of other neurological symptoms, including neck stiffness (*p* ˂ 0.01), and a CD4+ cell count between 100 and 199/µL (OR = 2.3 [95% CI: 1.04–5.1] *p* = 0.04) were associated with the presence of urine CrAg. A trend was also observed in the case of other neurological symptoms (OR = 4.4 [95% CI: 0.93–20.7] *p* = 0.06). The presence of respiratory symptoms such as cough was also more frequent in PLHIV with a CrAg detection (OR = 3.4 [95% CI: 1.02–11.4] *p* = 0.045).

## 4. Discussion

Opportunistic fungal diseases remain a health problem in Sub-Saharan Africa, notably in people living with HIV (PLHIV) [1]. In this study, the prevalence of *Cryptococcus neoformans* antigen in urine was determined in hospitalized patients in CHUL in order to facilitate the rapid management of CM.

Rapid and non-invasive tests for the diagnosis of diseases with clinical importance represent a good alternative in areas with insufficient or absent laboratory equipment and less experienced technicians. Although the sensitivity of the latex agglutination test was sometimes shown to be lower than lateral flow assays, and its performance could vary according to the different kits, CrAg detection is recommended by the World Health Organization and used according to the context [10,17,18]. Moreover, WHO recommends the CrAg detection for each PLHIV symptomatic or advanced disease, which is the case for more than 75% of the PLHIV hospitalized in the Infectious Disease department of the CHUL [10,19]. This ward is the reference center for HIV-positive inpatient management in the country, where more than 500 PLHIV are hospitalized each year, and more than 3000 are followed up by infectious disease physicians. In our context, there is no data on the burden of CrAg in PLHIV from Gabon, and CrAg detection is still not currently performed in HIV clinics. The Pastorex Crypto Plus (Bio-Rad) is the only CrAg test available in the country. Additionally, testing urine samples represents a non-invasive and simple methodology for a wide implementation of the WHO recommendations in settings where microscopes and well-trained lab technicians are lacking. Indeed, in most of the HIV treatment centers of urban and rural sites of Gabon, apart from malaria, HIV, and tuberculosis, the management of the other infectious diseases is often performed without biological confirmation. As hospitalized PLHIV often presents opportunistic infections like cerebral toxoplasmosis, CM, and/or central nervous system tuberculosis with overlapping symptoms, such tests would be useful for patient rapid screening and management of cryptococcosis [1,5]. Data on urine CrAg detection for CM were critically lacking. Its sensitivity was found to be either lower, similar, or even sometimes higher than the CrAg serum detection sensitivity [17]. The present results highlight the agreement between microscopic detection of *Cryptococcus.* sp in CSF and antigenuria, providing additional information on urinary CrAg agglutination assay utility as an alternative for other methods, such as serum CrAg detection for PLHIV with advanced disease. In Gabon, where microscopy and CSF culture are the main biological methods used for the diagnosis of CM, this is the first evaluation of a CrAg test. If confirmed in a larger population, the present results would serve as an advocacy for health authorities for a larger national survey that will combine the CrAg evaluation and comparison of serum and urinary methods for the estimation of the burden of CrAg among asymptomatic and symptomatic PLHIV. The aim is to implement routine CrAg detection in PLHIV to reduce CM morbidity and mortality in Gabon.

Cryptococcal antigenuria was found in almost one-quarter of the patients with neurological symptoms who were hospitalized in the CHUL. This high prevalence is certainly related to the inclusion criteria of the study participants. They all have neurological signs, and the majority were either at WHO clinical stages III or IV (80.0%) or with a CD4+ cell count below 200/mL (68.3%). The yeast load increased with the immunosuppression. Thus, they had several risk factors for CM and a high probability of being tested positive. Indeed, the CrAg burden was shown to be higher in hospitalized patients with advanced diseases, and a rate of 40% was found in Honduras [20]. In studies conducted in Tanzania and China, the prevalence of cryptococcal antigen varied from 4.4% to more than 30.0% [1,21,22,23]. The majority were women (71.5%), as reported in studies carried out in Ethiopia and Tanzania, with a prevalence of 54% and 61%, respectively [17,21]. In other settings, *Cryptococcus* antigen frequency was comparable between men and women [21,24,25]. In the present study, most of the PLHIV population was composed of women (>70%), as reported elsewhere [21,26,27,28]. The female predominance observed supports the tendency found in the general population infected by HIV in Gabon [13].

Almost all the patients (more than 80%) with *Cryptococcus*-positive antigenuria were under 50 years old. A high prevalence of CrAg in HIV-infected patients belonging to the same age group was previously described [21,22,29,30]. This may be related to the age of the patients, who were mostly under 55 years old, confirming that HIV infection particularly affects young adults (15 to 49 years) [7,31,32].

As far as marital status was concerned, singles (81.6%) predominated, while their prevalence was significantly lower among PLHIV in studies performed in Vietnam (39.2%) and Ethiopia (16.0%) [3,28]. The predominance of married participants in those studies could be explained by the acceptance (adherence and awareness) of their HIV status by these couples. Likewise, the predominance of singles may be due to the abandonment of men or women by their partners due to their HIV status.

Tuberculosis, toxoplasmosis, and candidiasis are among the most common associated diseases related to advanced HIV infection [33,34]. The main comorbidity reported in the present study was tuberculosis, as revealed in various studies [3,4,27]. Cryptococcal antigenemia may increase susceptibility to other illnesses, probably due to immune defects presumably related to genetic factors. Effective *Cryptoccocus neoformans* infection elimination and enhancement of patients’ survival are associated with an immune response based on pro-inflammatory cytokine production [7]. Immune response dysfunction predisposing to or provoked by antigenemia seems to increase the vulnerability to other opportunistic infections. Indeed, highlighting a link between prior TB and cryptococcosis suggests a common immune defect [35]. Individuals with CrAg are at higher risk of developing other AIDS-defining illnesses than CrAg-negative patients (RR, 2.69; 95% CI, 0.98–7.42; *p* = 0.05); various infections coexisting with cryptococcosis have also been reported [8]. Tuberculosis is the most common concomitant disease linked to advanced HIV infection, contributing to the development of Cryptococcosis [4,34,36]. In fact, 40% of included PLHIV with confirmed CM had confirmed tuberculosis in the present study. Most of the study participants received ART. Thus, the majority of CrAg+ patients (97.1%) were under antiretroviral therapy. Without treatment, the detection of CrAg heralds the onset of symptomatic CM, although individuals with antigenemia can remain asymptomatic for weeks to months before clinical meningitis occurs [3,26]. In South Africa, in patients initiating ART, the baseline cryptococcal antigenemia predicted the development of subsequent CM within 1 year after the start of their treatment, with 100% sensitivity and 96% specificity. Nevertheless, most of these patients had low CD4 cell count (˂200 cells). In the meantime, the positivity rate of *Cryptococcus* antigenuria was more elevated in patients with low CD4 cell count at an advanced stage of HIV infection. These results are consistent with previous studies reporting a high prevalence of CrAg-positive patients with CD4+ cell count below 200/mL [27]. These data support the need to introduce WHO’s recommendation of testing all PLHIV with CD4 T cell counts <100 cells/mm^3^ or with advanced disease [10,37]. The presence of cryptococcal antigenuria would also be a marker of poor clinical response and/or low CD4 cell count [21]. Urinary CrAg was mainly detected in PLHIV at WHO stages III and IV (80.0%), similar to reports from Vietnam and Tanzania [20,27].

All participants with urinary CrAg had at least two of the following usual meningitis clinical signs, as., headache, fever, neck stiffness, and psychological agitation. In addition to CD4 T cell count <100 cells/mm^3^, these signs and symptoms are predictive of cryptococcal meningitis [25,37,38]. In fact, studies conducted around the world have shown a relationship between clinical signs and symptoms (mainly headache and fever) and the detection of Cryptococcal antigenemia [1,3,21,22,25,27,38]. CrAg-positive patients complained of chronic headaches. The presence of headache multiplied by 3.2 the risk of developing cryptococcal antigenemia [39,40]. Complaints of chronic headaches in PLHIV at any stage of HIV infection should, therefore, draw attention to cryptococcosis infection. In other settings, the usefulness of CrAg RDT assays as first tests for the diagnosis of CNS cryptococcosis was also highlighted in patients with neurological symptoms [41,42,43]. The present data also suggest the usefulness of CrAg detection in CM-suspected patients with contra-indication of lumbar puncture or those living in remote settings. With this evidence-based information, the introduction of more sensitive rapid diagnosis assays, such as serum or urine lateral flow assays, could be considered either for the diagnosis of CNS cryptococcosis or for the screening of asymptomatic CrAg carriage in HIV clinics of Gabon.

Therefore, clinical signs combined with CrAg testing would contribute to a more frequent diagnosis and detection of cryptococcal meningitis in PLHIV. The aim of this study was not to discuss latex agglutination versus the LFA test as well as urine versus serum test sensitivity. Indeed, Cryptococcal antigen testing realized using either a latex agglutination test or a lateral flow assay is more sensitive than culture [5]. Thus, assuming that the Pastorex RDT has a non-negligible sensitivity for CrAg detection and would be more sensitive than clinical diagnosis in our context with frequent co-existence of several opportunistic infections in PLHIV with advanced disease, the CrAg detection would allow for the patient triage for a rapid management of cryptococcosis. A highly sensitive and specific antigen detection test in urine could also be used as an alternative to serum antigen detection for the asymptomatic or subclinical CM early diagnosis in immuno-compromised patients; this will allow for the initiation of fluconazole pre-emptive therapy [44]. Whatever the method used, CrAg should be introduced in Gabon, and its implementation should be supported by the national health policy.

This study has some limitations. First, its single-center design does not allow for the generalization of the results to the HIV population of Gabon. While the infectious disease ward is the reference center for HIV care and the single public hospitalization ward dedicated to HIV, data from the other public HIV clinics and private health structures should also be generated. Secondly, the high prevalence of antigenuria is probably more due to population inclusion criteria, such as the presence of neurological symptoms. Nevertheless, symptomatic patients tested negative, but due to this study’s objective and the design, their outcome is unknown. Other hospitalized PLHIV without neurological signs may not have been included, although they could be at the early stage of the infection. Third, urinary CrAg detection and agglutination assays were sometimes found to be less sensitive than lateral flow assay in serum samples for early CM diagnosis. Nevertheless, as for other diseases, other non-gold standard tests with acceptable performances could be used in the absence of the standard method. Moreover, antigenuria is a good biomarker of pathogen diffusion. From a methodological point of view, testing symptomatic PLHIV with a high probability of CM is the first step in the process of CrAg detection introduction for the routine screening for cryptococcosis in PLHIV in Gabon. Indeed, if a test fails to detect the CrAg in symptomatic patients in whom the level of antigens is often very high, then it cannot be very useful for the detection of CrAg in asymptomatic patients. In the absence of lateral flow assays in Gabon, the Pastorex RDT, which does not require any additional equipment than that used for the routine biological follow-up of PLHIV in HIV clinics, would serve as an alternative. Moreover, urine collection can be performed by any trained health community worker in the field for further analysis in the closest HIV clinic.

The results presented here provide important information that was lacking in Gabon and that can be considered as a basis for further investigations.

## 5. Conclusions

The prevalence of Cryptococcal antigenuria is high in hospitalized PLHIV with neurological symptoms, indicating a non-negligible prevalence of CM in this population. Antigenuria was found to be a good marker of CM in the study population, and it was associated with low CD4+ cell count, WHO stage III/IV, and tuberculosis. CrAg screening should be introduced in the routine care of PLHIV with advanced disease in Gabon, as recommended by WHO.

## Figures and Tables

**Table 1:** Distribution of Cryptococcus urinary antigen (CrAg/urine, N = 142).

Socio-Demographic	All Patients N (%) N = 142	CrAg Positive N (%) * N = 35	CrAg Negative N (%) * N = 107	*p* Value
Gender				0.45
Female	102 (71.8)	25 (71.5)	77 (72.0)	
Male	40 (28.12)	10 (28.5)	30 (28.0)	
Age (year)				0.95
<55 years	123 (86.6)	29 (82.8)	94 (87.8)	
>55 years	19 (13.4)	6 (17.2)	13 (12.2)	
Matrimonial status				
Single	116 (81.6)	28 (80.0)	88 (82.2)	
Married	22 (15.3)	7 (20.0)	15 (14.0)	
Widowed	4 (2.1)	0 (0.0)	4 (3.7)	
Occupation				
Worker	61 (42.9)	15 (42.8)	46 (42.9)	
Unemployed	48 (33.8)	11 (31.4)	37 (34.5)	
Manager	33 (23.3)	9 (25.7)	24 (22.4)	
Comorbidity				0.001
Tuberculosis	26 (18.3)	14 (40.0)	12 (11.2)	
Hypertension	1 (0.7)	0 (0.0)	1 (0.9)	
Diabetes	1 (0.7)	0 (0.0)	1 (0.9)	
HIV Diagnosis				
1 year	36 (25.3)	5 (14.3)	31 (28.9)	
2–5 years	37 (26.1)	12 (34.3)	25 (23.4)	
6–10 years	32 (22.5)	11 (31.4)	21 (19.6)	
>10 years	37 (26.1)	7(20.0)	30 (28.0)	
Antiretroviral treatment (ART)				0.33
Yes	123 (86.6)	34 (97.1)	89 (83.2)	
No	19 (13.4)	1 (2.9)	18 (16.8)	

* % refers to the frequency of individuals among CrAg positive or negative individuals according to each socio-demographic variable.

**Table 2:** PLHIV clinical and immunological characteristics according to the presence of urine CrAg.

	All Patients N (%)	CrAg Positive N (%) N = 35	CrAg Negative N (%) N = 107	*p* Value
WHO Clinical stages				0.45
I	55 (38.7)	4 (11.4)	51(47.6)	
II	13 (9.1)	3 (8.6)	10 (9.4)	
III + IV	74 (52.2)	28 (80.0)	46 (43.0)	
Symptoms				
Fever	123 (86.6)	32 (91.4)	91 (85.0)	0.28
Headache	115 (80.9)	32 (91.4)	83 (77.6)	0.07
Altered consciousness	7 (4.9)	6 (17.2)	1 (0.9)	0.001
Vomiting	7 (4.9)	4 (11.4)	3 (2.8)	
Other Neurological symptoms	7 (4.9)	4 (11.4)	3 (2.8)	0.04
Neck Stiffness	7 (4.9)	7 (100.0)	0 (0.0)	0.002
Seizures	7 (4.9)	5 (14.3)	2 (1.9)	0.003
Respiratory symptoms	12(8.4)	6 (17.2)	6 (5.6)	0.03
CD4+ cell count (cell/µL)				0.015
<100	27 (19.1)	5 (14.3)	22 (20.6)	
[100–199]	70 (49.2)	20 (57.2)	50 (30.0)	
[200–349]	19 (13.4)	8 (22.8)	11 (10.3)	
[350–499]	15 (10.6)	2 (5.7)	13 (8.4)	
>499	11 (7.7)	0 (0.0)	11 (14.0)	

Frequency of CrAg Positive Test

## Data Availability

The original contributions presented in the study are included in the article, further inquiries can be directed to the corresponding author.
